# Penile gangrene due to improper application of a condom catheter: a case report

**DOI:** 10.1186/s13256-023-03771-x

**Published:** 2023-01-31

**Authors:** Kays Chaker, Yassine Ouanes, Makram Rinchi, Nizar Cherni, Mokhtar Bibi, Kheireddine Mrad Dali, Yassine Nouira

**Affiliations:** Department of Urology, La Rabta Hospital, Bab Saadoun, Tunis, Tunisia

**Keywords:** Penis, Gangrene, Condom catheter, Penectomy

## Abstract

**Background:**

Fournier’s gangrene is a rare, fulminant, and usually localized necrotizing soft tissue polymicrobial infection of the perineum, with occasional extension up to the abdominal wall.

**Case presentation:**

We describe an unusual case of penile gangrene in a 64-year-old Tunisian man suffering from urinary incontinence secondary to cerebrovascular accident. Gangrene developed due to continuous tourniquet effect on the penis caused by a condom catheter. Although source control was achieved with aggressive debridement, careful wound care, and wide-spectrum antibiotherapy, the patient died due to septic shock.

**Conclusion:**

Use of condom catheters is not without complications. Careful placement, strict hygiene, and regular monitoring of the local condition are necessary.

## Background

Condom catheters are external urinary drainage devices mainly applied in bedridden and incontinent patients [1]. These catheters are not completely without risk. Multiple complications have been described including maceration, ulceration, allergic reactions, urinary tract infections, localized ischemia, and gangrene [2, 3]. We report a case of total gangrene of the penis due to an improperly applied condom catheter.

## Case observation

**Fig. 1:**
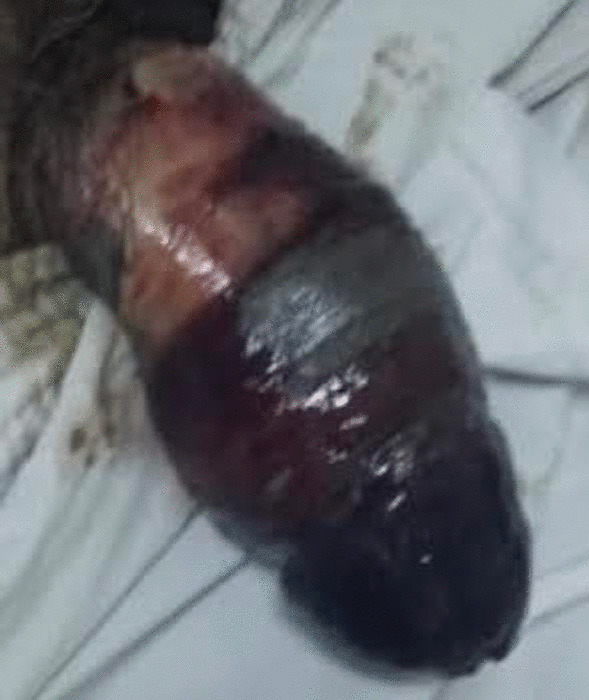
Penile gangrene due to condom catheter

**Fig. 2:**
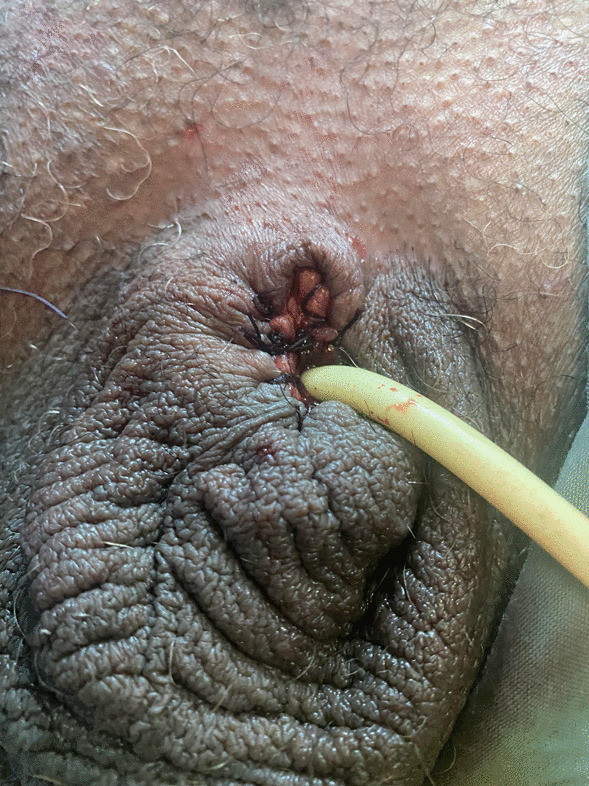
Postoperative indwelling cystostomy

A 64-year-old Tunisian man with history of hypertension and type 2 diabetes was initially managed for cerebrovascular accident due to right parieto-occipital infarction. After stabilization, he was put on anticoagulants and was discharged from the hospital with a condom catheter. Ten days later, he presented with fever and pain, with brownish black discoloration of the penis. On general physical examination, the patient was febrile. On examination, a constricting band (the adhesive tape used to secure the condom catheter) was seen at the base of the penis. The distal penis was engorged and gangrenous with a clear line of demarcation (Fig. 1). No sensations were present, and pulsations distal to the constriction were absent. There were no other foci of infection in the genitoperineal area. The prostate gland was normal on rectal examination. Laboratory examination revealed white blood cell count of 19,000/mm^3^ with left shift, C-reactive protein of 310 mg/L, and random blood sugar of 450 mg/dL. Blood urea and serum creatinine were within normal limits. Purulent material discharge from penis was sent for culture. Blood, urine, and pus cultures were obtained. The patient was started on broad-spectrum antibiotics (tazobactam and amikacin), and fluid resuscitation was initiated. Emergent exploration and debridement were performed. Along the necrosis margin, a circular incision was made and the pus drained and sampled for culture. Corpora cavernosa and urethra were necrotic. Penectomy, urethrectomy, and debridement of the necrotic tissues were performed. An indwelling cystostomy catheter was placed (Fig. 2). Penile swab culture resulted in *Klebsiella pneumoniae* spp. Although source control was achieved with aggressive debridement, careful wound care, and wide-spectrum antibiotherapy, the patient died due to septic shock.

## Discussion

Fournier’s gangrene is a necrotizing fasciitis of the genital area that can extend to the perineum and the anorectal region [4]. The majority of cases are polymicrobial and require emergent debridement and wide-spectrum antibiotic treatment [4]. Colorectal sources (30–50%), urogenital sources (20–40%), cutaneous infections (20%), and local trauma are the main causative agents for Fournier’s gangrene [5]. It is usually seen in immunocompromised patients such as those with diabetes or end-stage renal disease [5]. Penile gangrene is a rare entity that can manifest in two forms: dry or wet, each of which requires different processing [4]. Penile dry gangrene is the result of ischemia and is most commonly associated with long-standing diabetes mellitus and end-stage renal disease leading to secondary hyperparathyroidism [4]. This situation will lead to calciphylaxis of the penile arteries and thus reduced blood flow [4]. Less frequent causes include tourniquet effect, priapism, venous thrombosis, anticoagulant treatment, and injection of heroin in the femoral vessels, or penile prosthesis [4]. Wet gangrene or “Fournier’s gangrene” is a synergistic necrotic fasciitis of genitalia, perineum, and abdominal wall. It is a rare, rapidly progressing, and potentially fatal soft tissue infection, first described by J.A. Fournier, who described 5 cases of penis and scrotum gangrene without obvious cause [6]. Fournier’s gangrene is rarely truly idiopathic. However, recent studies indicate that FG seems to be more frequent in diabetes mellitus (most common), obesity, cancers, alcohol abuse, advanced age, poor hygiene, malnutrition, heart and peripheral arteries diseases, liver disease, chronic renal failure, HIV infection, and immunodeficiency. Furthermore, local trauma, periurethral urine leak, perineal surgery, paraphimosis, and penile sexual trauma have been implicated [7]. Both aerobic and anaerobic microorganisms may be implicated in the infection, and cultures usually reveal *Escherichia coli* [7]. The time between diagnosis and treatment greatly affects morbidity and mortality, and it can quickly progress to sepsis [4]. This is why it remains a life-threatening disease. The cornerstones of management are urgent patient resuscitation, broad-spectrum antibiotic therapy, surgical debridement, and reconstructive surgeries. Parenteral broad-spectrum antibiotics are required, including triple therapy: third-generation cephalosporins or aminoglycosides, plus penicillin and metronidazol, then adjusted according to the result of cultures. Early surgical debridement, under general or spinal anesthesia, is always recommended where necrotic tissue must be removed until the wound bed is clean [4]. Almost all cases had good recovery after surgery and satisfactory reconstruction, using either skin graft or local scrotal flap [7]. But in our case, the septic complication was fatal.

## Conclusion

Healthcare professionals should be aware that improperly applied condom catheters can lead to penile gangrene, a rare but serious condition. Prevention is the key, by maintaining strict hygiene and frequent monitoring of the device.

## Data Availability

The datasets are available from the corresponding author on reasonable request.
